# Supplementary material for: β-Cyclodextrin-Encapsulated Rhodamine Derivatives Core–Shell Microspheres—Based Fluorescent Sensor for Au3+ and Template for Generating Microplates of Gold
Source: Micromachines (Basel). 2023 Jul 18;14(7):1443. doi: 10.3390/mi14071443 (PMC10384120; doi:10.3390/mi14071443)
Supplement: Supplementary file 1 [file micromachines-14-01443-s001.zip › micromachines-2465537-supplementary.pdf]

Supplementary Information

β-Cyclodextrin-Encapsulated Rhodamine Derivatives Core–Shell Microspheres—Based Fluorescent Sensor for Au^{3+} and Template for Generating Microplates of Gold

Maniyazagan Munisamy [1,2,†], Balamurugan Rathinam [3,†], Esakkimuthu Shanmugasundaram [1], Vigneshkumar Ganesan [1], Vimalasruthi Narayanan [1] and Suganya Bharathi Balakrishnan [1], Selvam Kaliyamoorthy [4,*] and Stalin Thambusamy [1,*]

1 Department of Industrial Chemistry, School of Chemical Sciences, Alagappa University, Karaikudi 630003, India; manichemist@gmail.com (M.M.); asmuthu92@gmail.com (E.S.); gvkumar.chemist@gmail.com (V.G.); annamsruthi2014@gmail.com (V.N.); sbsuganyaa@gmail.com (S.B.B.)
2 Department of Nanotechnology and Advanced Materials Engineering, Sejong University, Seoul 05006, Republic of Korea
3 Department of Chemical and Materials Engineering, National Yunlin University of Science and Technology, Yunlin 64002, Taiwan; balar@yuntech.edu.tw
4 Department of Chemistry for Materials, Graduate School of Engineering, Mie University, Tsu 514-8507, Japan
* Correspondence: chemsel@gmail.com (S.K.); stalin.t@alagappauniversity.ac.in (S.T.)
† These authors contributed equally to this work.

Citation: Munisamy, M.; Rathinam, B.; Shanmugasundaram, E.; Ganesan, V.; Narayanan, V.; Balakrishnan, S.B.; Kaliyamoorthy, S.; Thambusamy, S. β-Cyclodextrin-Encapsulated Rhodamine Derivatives Core–Shell Microspheres—Based Fluorescent Sensor for Au^{3+} and Template for Generating Microplates of Gold. *Micromachines* **2023**, *14*, 1443. https://doi.org/10.3390/mi14071443

Academic Editor: Wing-Cheung (Roy) Law

Received: 7 June 2023
Revised: 12 July 2023
Accepted: 14 July 2023
Published: 18 July 2023

Copyright: © 2023 by the authors. Licensee MDPI, Basel, Switzerland. This article is an open access article distributed under the terms and conditions of the Creative Commons Attribution (CC BY) license (https://creativecommons.org/licenses/by/4.0/).

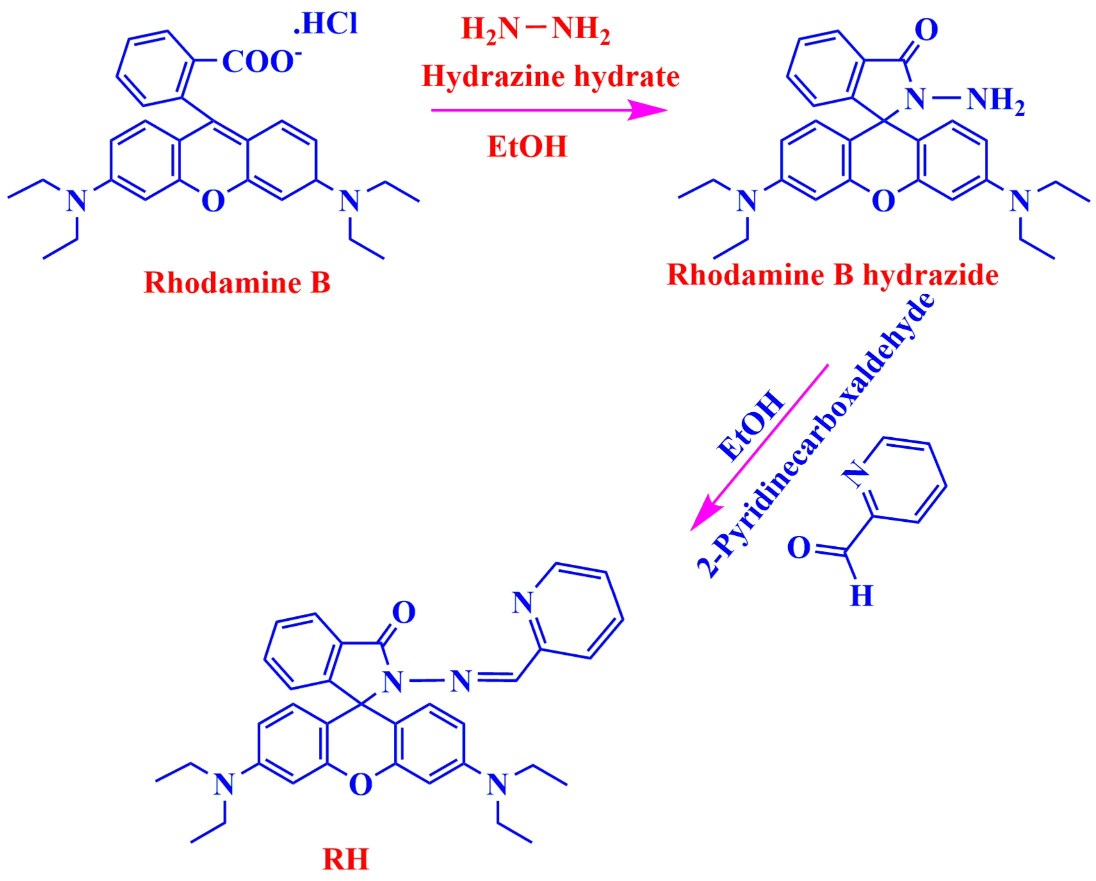

Scheme S1. Synthetic scheme for fluorescent probe RH.

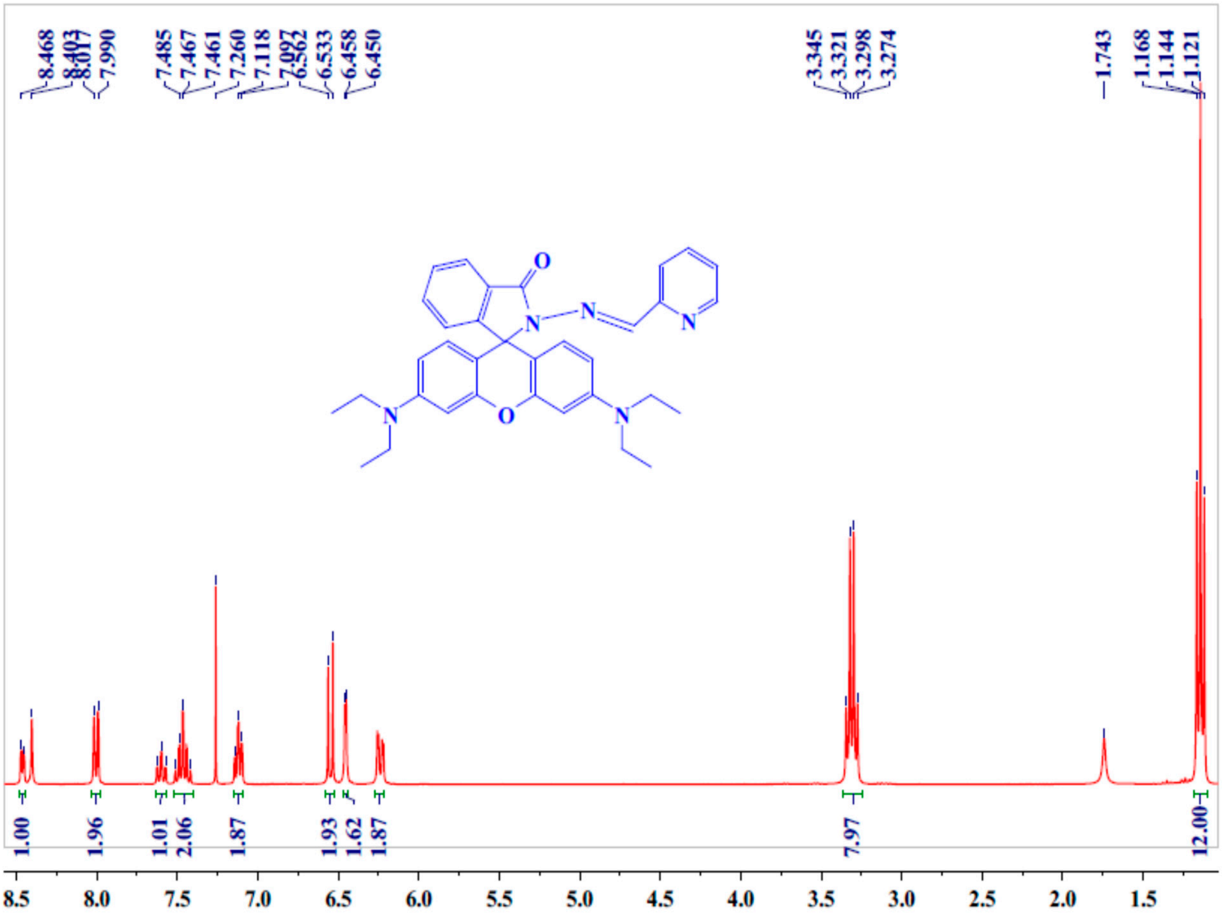

Figure S1. ^{1}H NMR ($CDCl_3$) of RH.

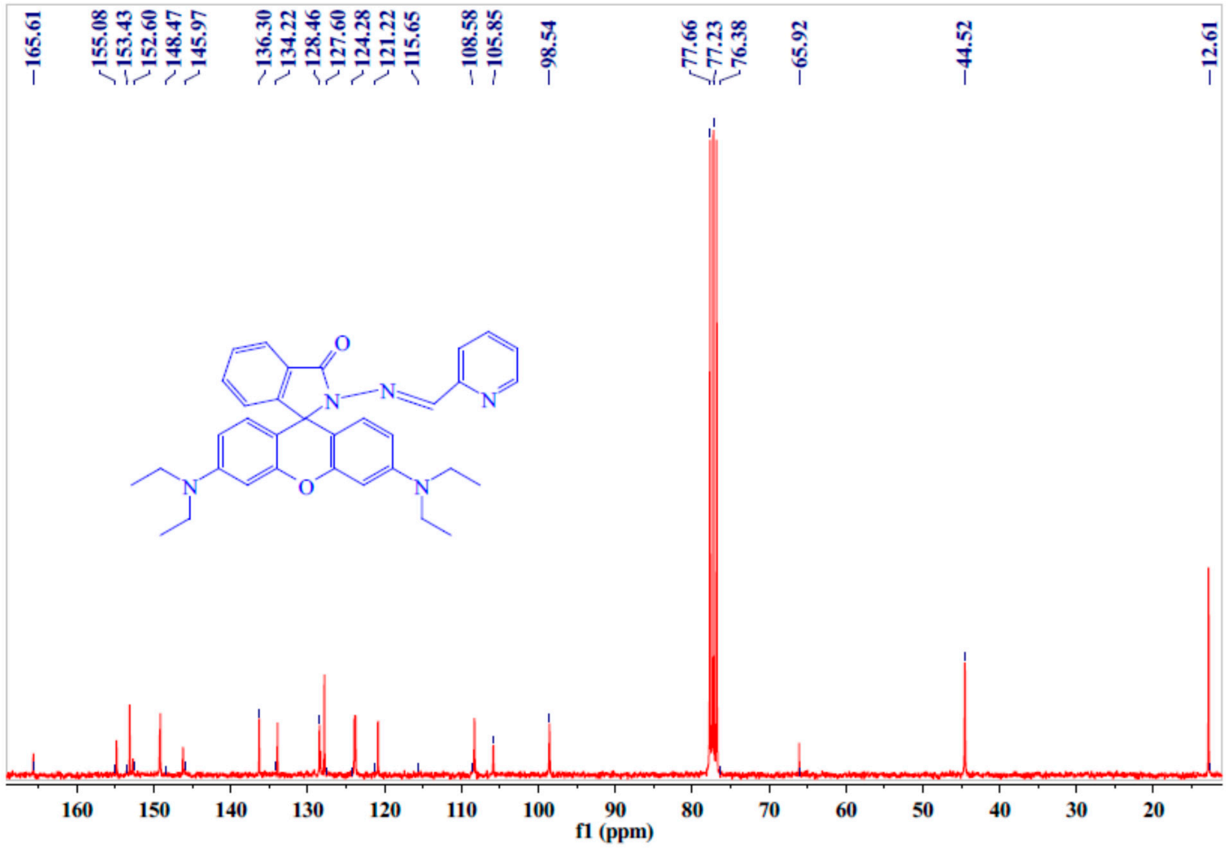

Figure S2. ^{13}C NMR of RH($CDCl_3$).

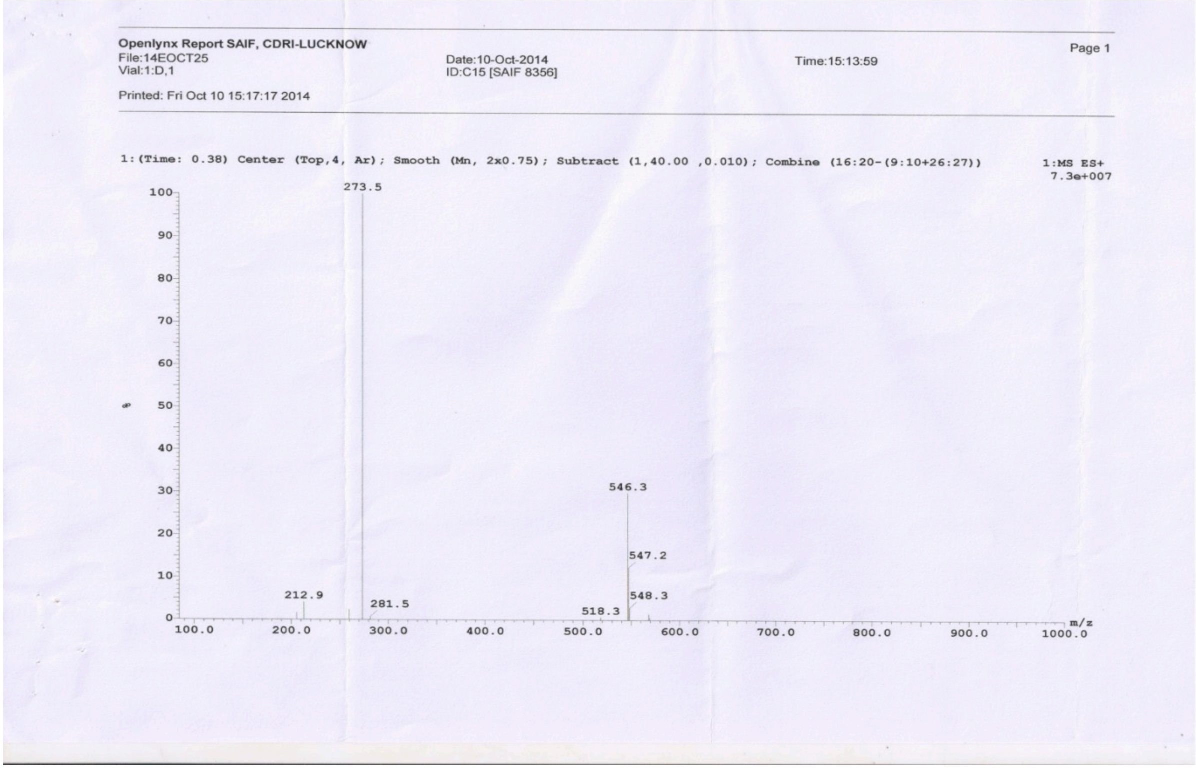

Figure S3. The ESI mass spectra of RH.

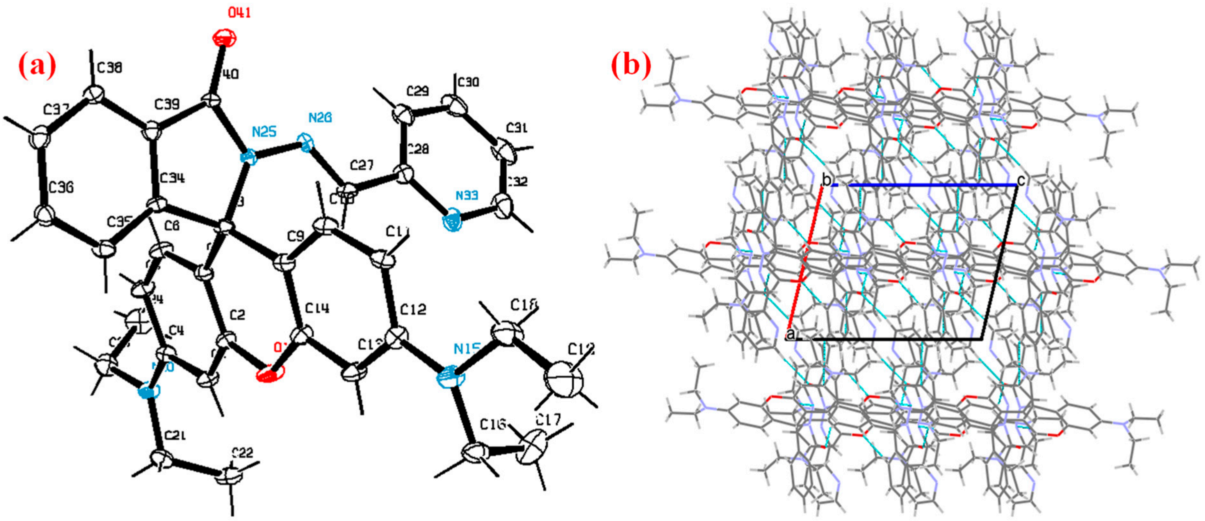

Figure S4. (a) X-ray crystal structure of RH showing displacement ellipsoids drawn at the 30% probability level. Hydrogen atoms have been omitted for clarity. (b) The crystal packing of RH. Intermolecular interactions have been omitted for clarity.

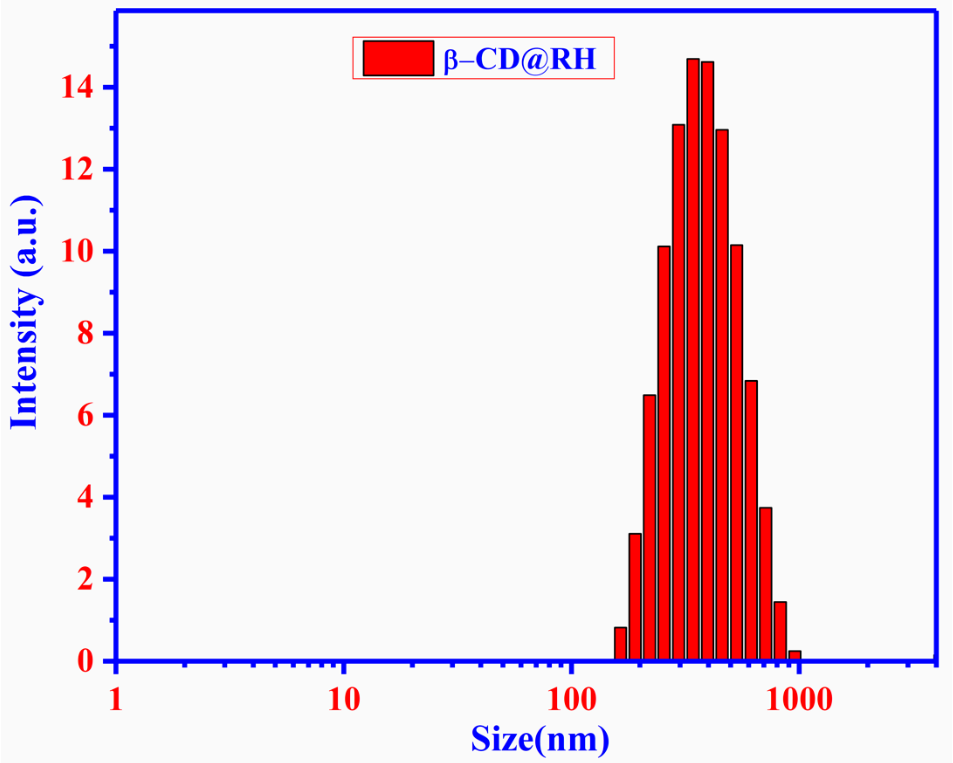

Figure S5. Dynamic laser scattering of β-CD@RH microspheres.

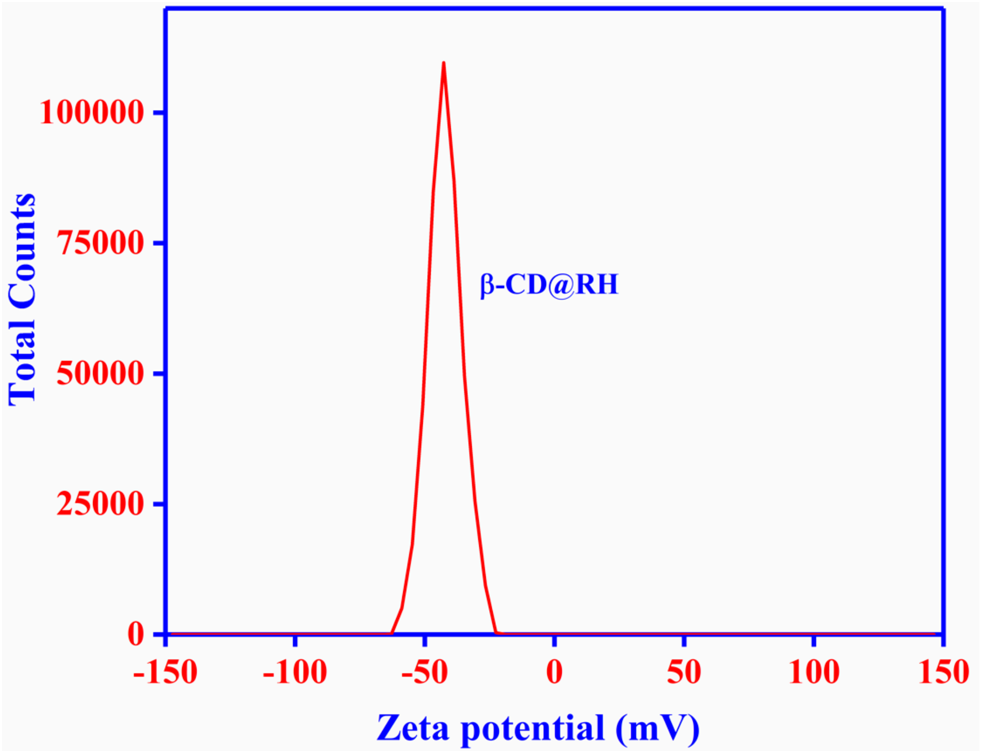

Figure S6. Zeta potential of β-CD@RH microspheres.

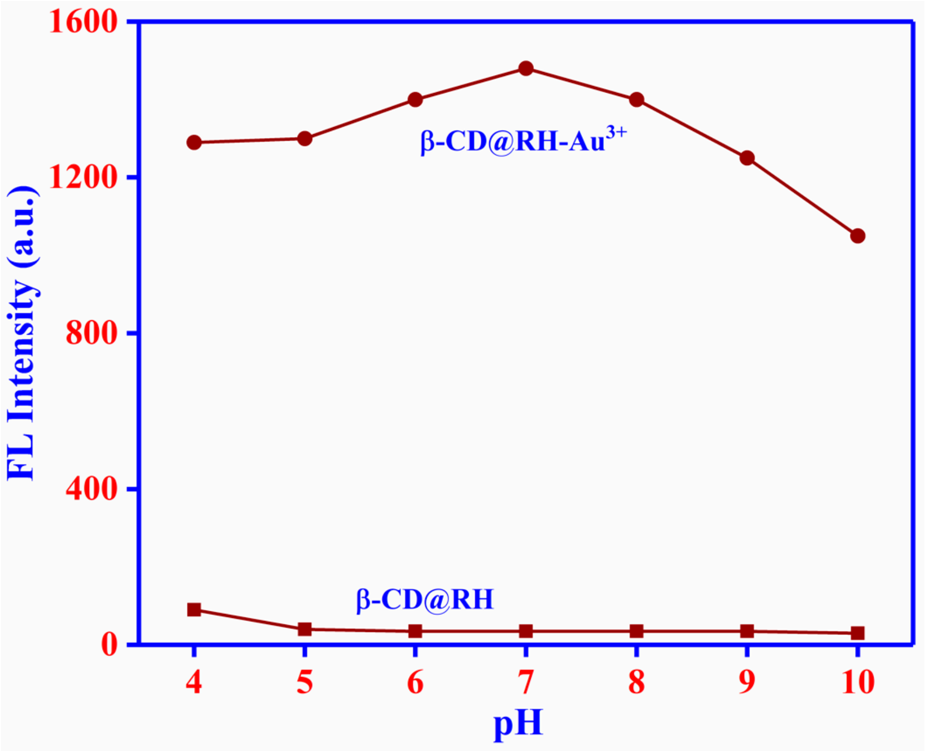

Figure S7. Fluorescence response of β-CD@RH in absence and in presence of Au^{3+} ions in water media at different pH (λ_{em} = 582 nm).

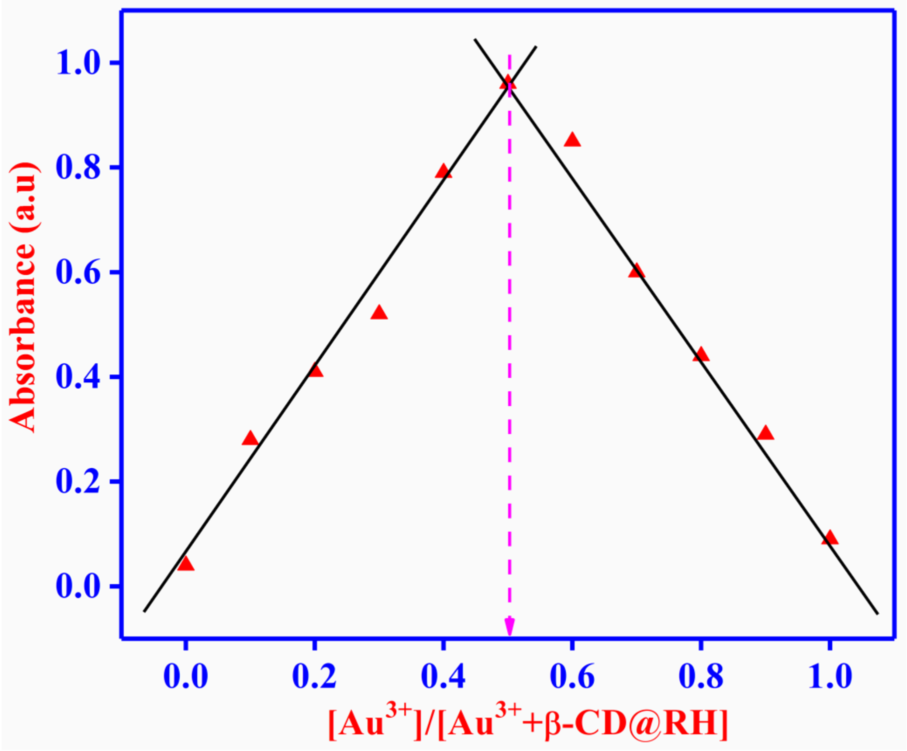

Figure S8. Job's plot from the absorption data for stoichiometry determination between β-CD@RH and Au^{3+} ions in water at 25 ˚C at λ_{abs} = 556 nm.

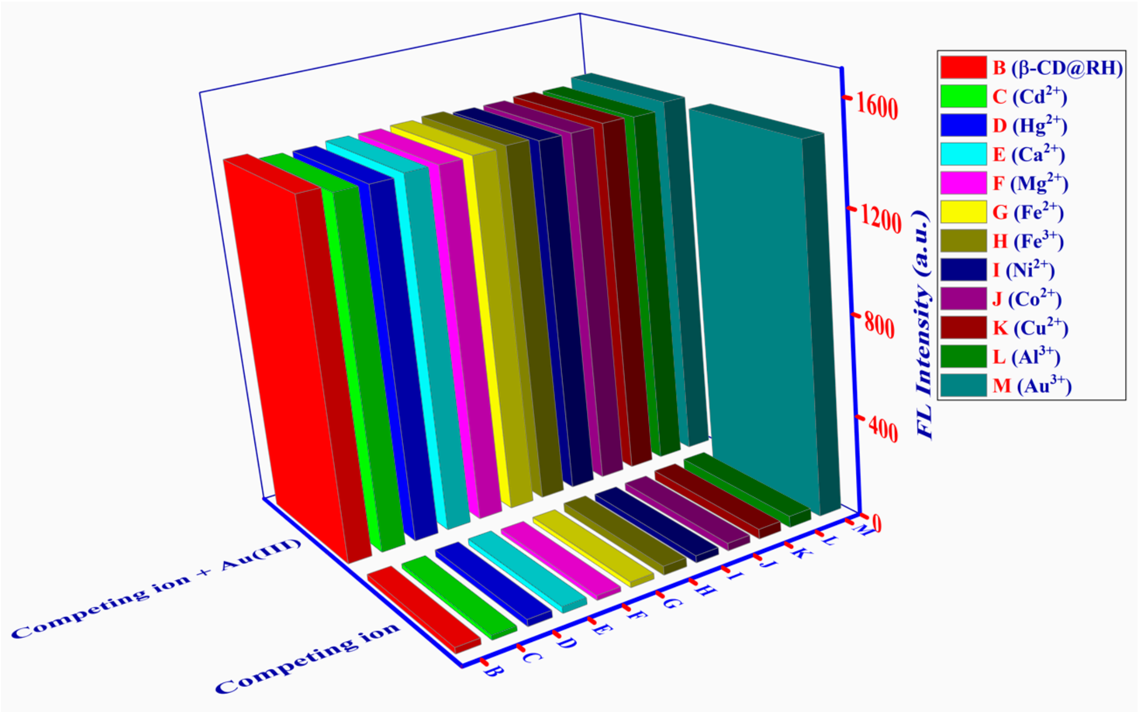

Figure S9. Results of the competition experiments between Au^{3+} and selected metal ions. The free β-CD@RH concentration was set at 10 μM, and the excitation was at 556 nm with a slit width of 5.0 nm.

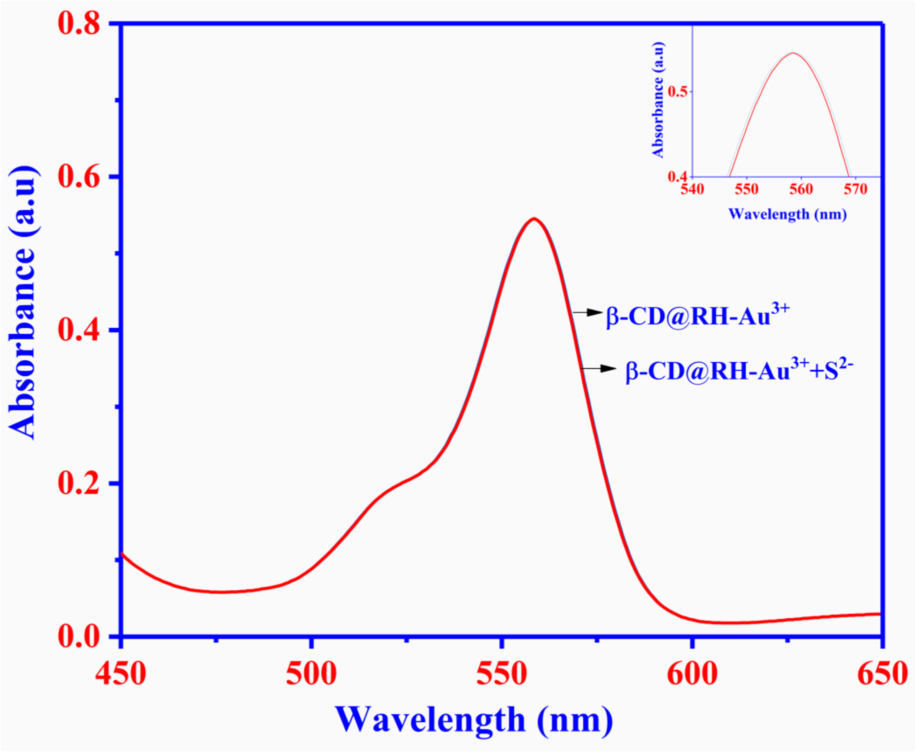

Figure S10. UV−vis titration spectra of β-CD@RH (10 μM) with 5 equiv. of Au^{3+} upon addition of sodium sulfide (30 μM) in water.

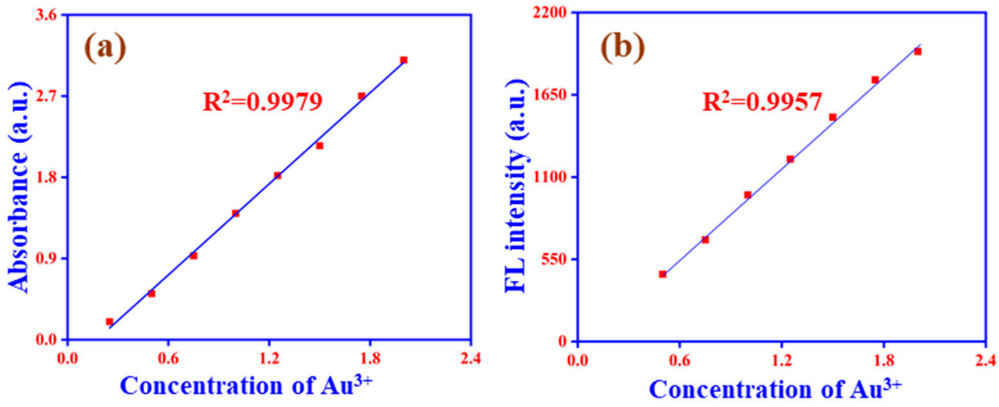

Figure S11. (a) Concentration variation of Absorbance intensity Vs. Concentration of Al^{3+} (0 to 2 equiv.). (b) Concentration variation of FL intensity Vs. Concentration of Al^{3+} (0 to 2 equiv.).

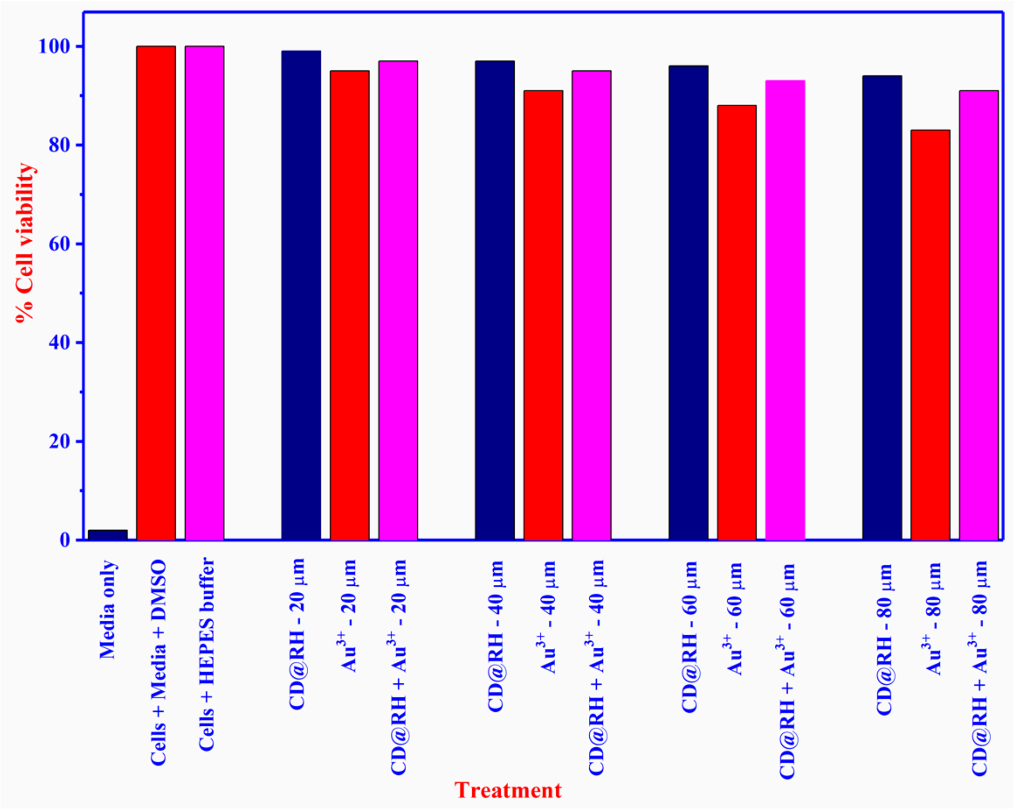

Figure S12. Cytotoxic effect of β-CD@RH, Au^{3+} and β-CD@RH-Au^{3+} in Mesenchymal Stem Cells (MSCs) incubated for 6 h by MTT assay. Results are expressed as mean of three independent experiments.

Table S1. Crystal data and details of refinements for **RH**

	RH
Empirical Formula	$C_{34}H_{35}N_5O_2$
Formula Weight	545.67
CCDC No	1440773
Crystal System	Monoclinic
Space group	$P2_{1/c}$
a(Å)	9.3925 (3)
b (Å)	25.6728 (10)
c(Å)	12.0364 (4)
β (°)	103.692 (2)
Density(mg/m^3)	1.285
Volume(Å^3)	2819.88 (17)
Temperature,K	296
Z	4
F(000)	1160
Θ range (deg)	4.2 to 64.4
Collected reflections	14441
Independent reflections	4549
Goodness-of-fit	1.07
R1[I>2.0σ(I)]	0.074
wR1[I >2.0σ(I)]	0.247
